# Supplementary material for: Long ncRNA A-ROD activates its target gene DKK1 at its release from chromatin
Source: Nat Commun. 2018 Apr 24;9:1636. doi: 10.1038/s41467-018-04100-3 (PMC5915440; doi:10.1038/s41467-018-04100-3)
Supplement: Supplementary file 1 — Supplementary Information [file 41467_2018_4100_MOESM1_ESM.pdf]

Long ncRNA A-ROD activates its target gene DKK1 at its release from chromatin

Ntini et al.

Supplementary Information contains:

Supplementary Figures 1-9

Supplementary Tables 1-2

Supplementary Figure 1

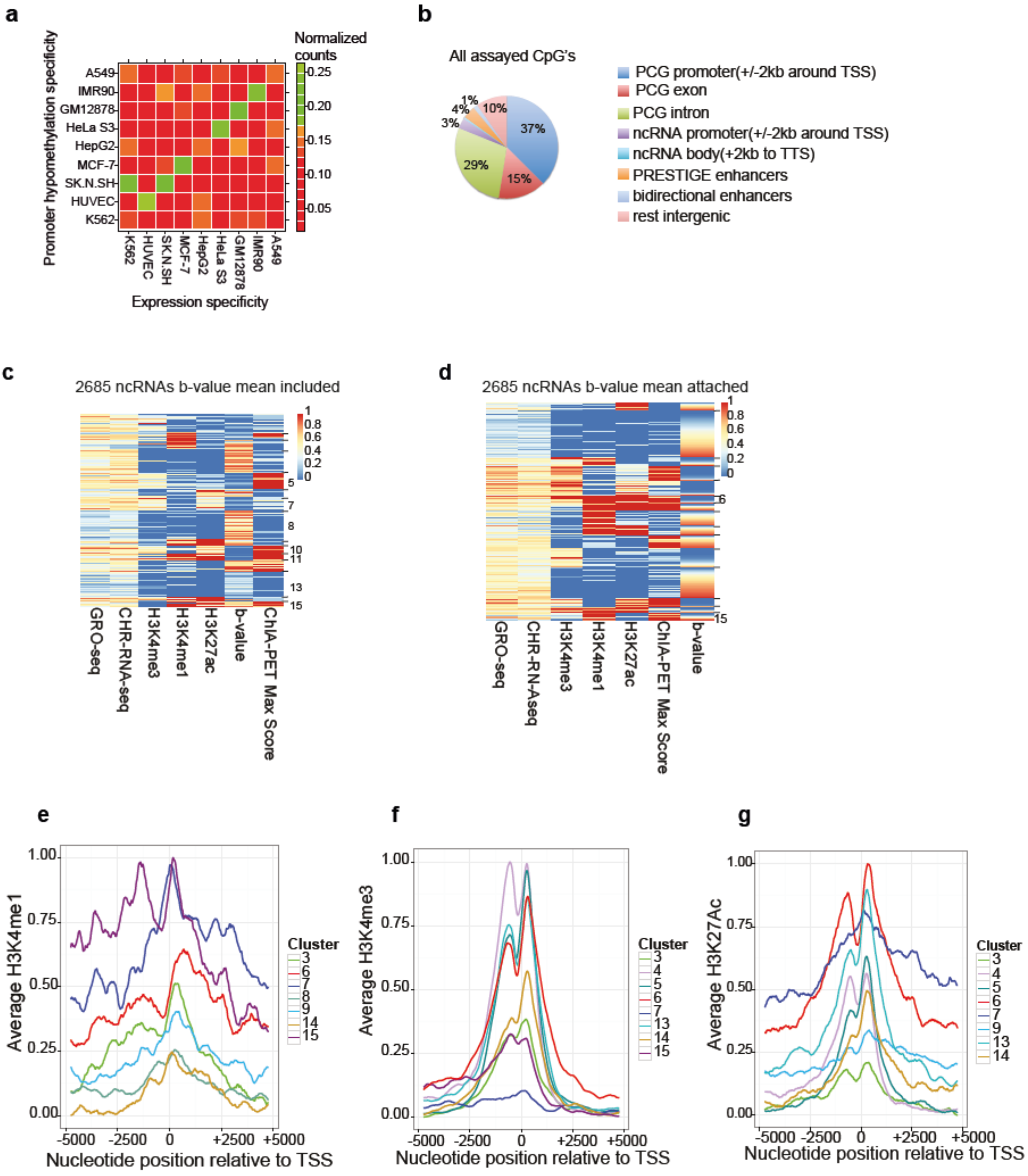

Supplementary Figure 1: Grouping of long ncRNAs based on expression and epigenetic marks

(a) Heatmap showing correspondence (normalized counts; observed/expected counts for each cell-type combination) between long ncRNA expression specificity (extracted from ENCODE available nuclear polyA+ RNA-seq data) and associated promoter hypomethylation cell-type specificity (extracted from the ENCODE Methyl 450K Array b-values, Methods). **(b)** Pie chart of the genomic distribution of SureSelect assayed CpG's common for 0 h (EtOH control) and 40 min estradiol (E2) treatment; in total 3,525,513 CpG's with an applied read coverage cutoff of 5. Protein coding gene (PCG) and long ncRNA promoters are here defined as ±2kb intervals around the transcription start site (TSS). PRESTIGE enhancers are from[1]. Putative bidirectional enhancers (±1kb extended) are from[2]. **(c-d)** Related to main Fig. 1b: **(c)** K-means clustering of 2,685 long ncRNAs including expression (GRO-seq[3] and chromatin-associated RNA-seq), histone mark signal values, ChIA-PET maximum interaction scores and SureSelect assayed DNA methylation (promoter b-value mean). **(d)** This heatmap is the result of the following analysis: k-means clustering was initially done for the 4,467 long ncRNAs without DNA methylation as a clustering parameter (Fig. 1b) and then the promoter b-value mean was attached for the entries with assayed DNA methylation (n = 2,685) producing the heatmap shown here (entries have been ordered within each cluster according to increasing DNA methylation). **(e-g)** Positional distribution of histone marks around the transcription start site (TSS) of the clustered long ncRNAs of Fig. 1b. Average histone mark signal (IP/Input) for **(e)** H3K4me1, **(f)** H3K4me3 and **(g)** H3K27Ac was smoothened over a 500 bp sliding window average (sliding step 1) ±5kb around the TSS of the clustered long ncRNAs followed by min-max rescaling for all.

Supplementary Figure 2

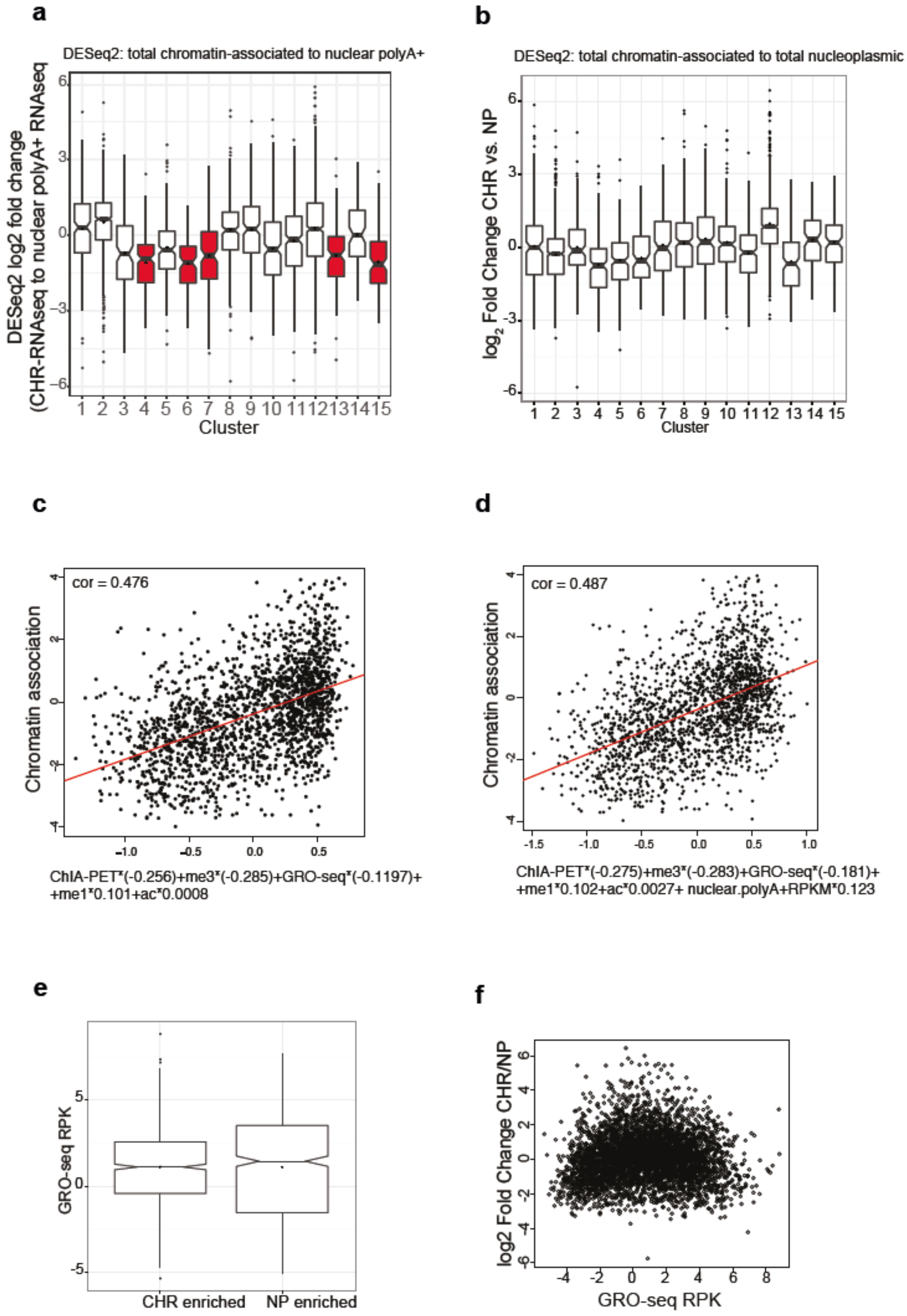

Supplementary Figure 2: Differential chromatin-association of long ncRNAs

(a) DESeq2 generated log2 fold changes in expression using total chromatin-associated versus nuclear polyA+ RNA-seq (ENCODE), for the clustered long ncRNAs of Fig. 1b. (See also Fig. 1d-e). **(b)** DESeq2 generated log2 fold changes in expression using total chromatin-associated versus nucleoplasmic RNA-seq, for the clustered long ncRNAs of Fig. 1b. (See also Fig. 1d-e). Significantly nucleoplasmic-enriched long ncRNAs (at *DESeq2* p-adjusted < 0.1) are significantly enriched in clusters 4, 5, 6 and 13 (Fisher's exact test p-value = [5.676e-12, 1.026e-12, 2.653e-06, 1.089e-07] and odds ratio = [2.53, 2.45, 2.35 and 2.36], respectively). **(c)** Linear regression analysis by incorporating the parameters ChIA-PET interaction score, GRO-seq RPKM, H3K4me3, H3K4me1 and H3K27ac, in the prediction of chromatin-association of long ncRNAs. The generated numeric coefficients for each variable are included in the type below the scatterplot. The red line represents the linear regression model fit (correlation = 0.476, p-value < 2.2e-16). The variables ChIA-PET score and H3K4me3 produce the highest negative coefficients in determining the fit (-0.256 and -0.285, respectively, at p-value < 2.2e-16). **(d)** Same as in c) but with one additional variable included, that is nuclear polyA+ RPKM. **(e)** Boxplot showing expression (GRO-seq reads per kb, RPK) of significantly chromatin enriched (CHR enriched, n = 847) and significantly nucleoplasmic enriched (NP enriched, n = 719) long ncRNAs, at *DESeq2* p-adjusted < 0.1 and a cutoff of *DESeq2* log2 fold-change CHR/NP set at 1, i.e. at least two fold enrichment either in chromatin or nucleoplasm. **(f)** Scatterplot of *DESeq2* generated log2 fold changes CHR/NP and GRO-seq RPK for all assayed long ncRNAs.

Supplementary Figure 3

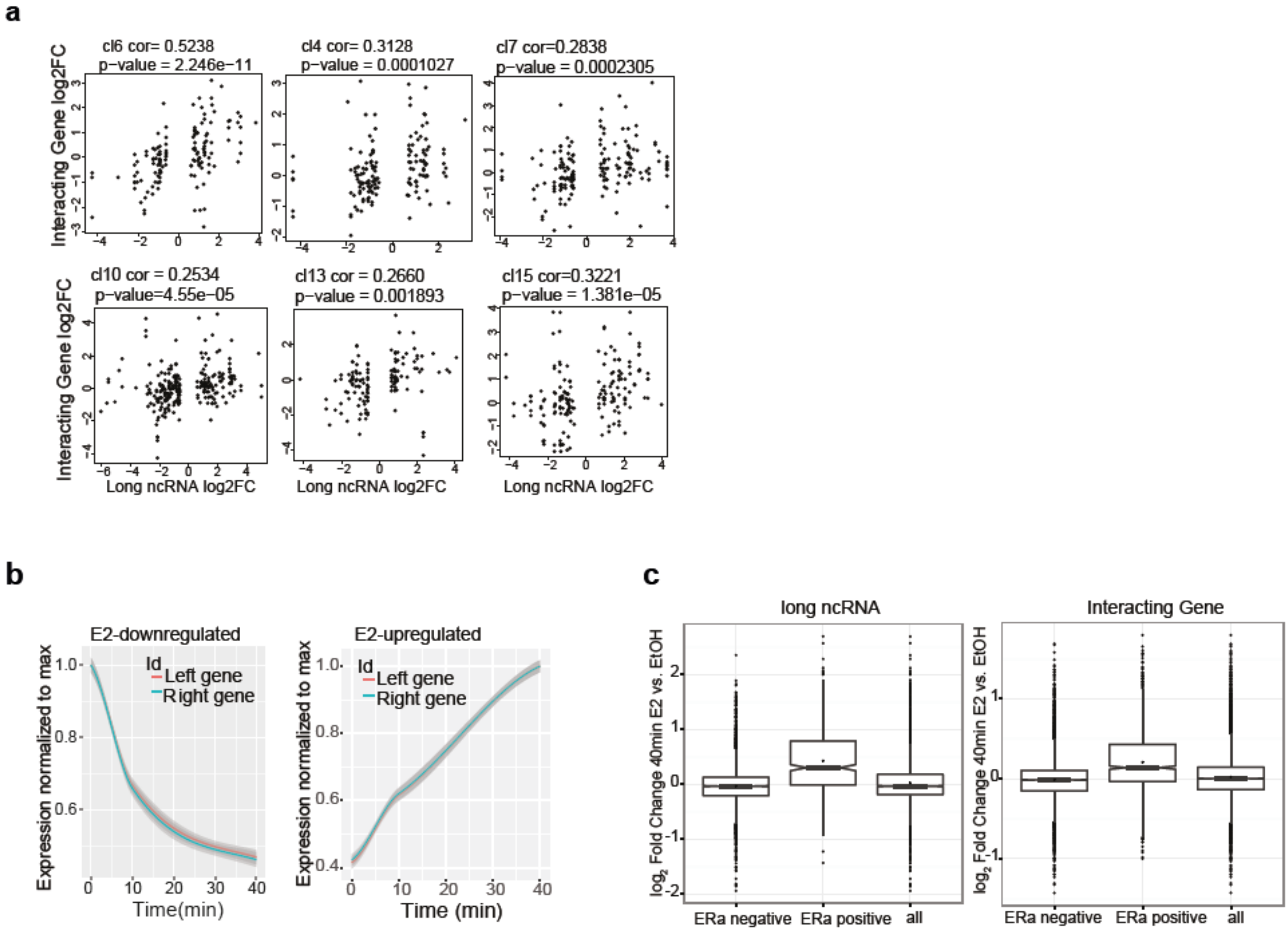

Supplementary Figure 3: Coordinated expression responses of ChIA-PET interacting pairs

(a) Scatterplots showing correlation of E2-mediated log2 fold changes in expression (40 min E2 treatment vs. EtOH control; GRO-seq[3]) for long ncRNAs of clusters 6, 4, 7, 10, 13, 15 (Fig. 1b) and ChIA-PET identified interacting genes. **(b)** E2-mediated time response of downregulated (left panel) and upregulated (right panel) randomly selected ChIA-PET interacting gene-gene pairs (with a ChIA-PET score > 200). GRO-seq RPKM from 0, 10 and 40 min of E2 treatment were normalized to maximum expression i.e. to the 0 min in the case of downregulated pairs (left panel) and to the 40 min time point in the case of upregulated pairs (right panel). Fitted regression model lines (loess curves) are shown and the 95% confidence interval is grey shaded. The analysis was ran several times (> 10) with different pools of randomly sampled ChIA-PET interconnected gene-gene pairs, producing similarly overlapping time responses. **(c)** Boxplot showing *DESeq2* generated regularized

*log*2 fold changes in expression of 40 min E2 treated MCF-7 cells versus control, using GRO-seq[3], for long ncRNAs (left) and their ChIA-PET identified interacting genes (right), sub-categorized as either ERa-positive or ERa-negative, based on the presence or not of ERa binding sites (ChIP-seq peaks) +/-10 kb around the transcription start site (TSS). (ERa ChIP-seq data are from[4] (GSM365926). For the 4,467 long ncRNAs (left): 513 have ERa +/-10 kb around the TSS (ERa-positive), and of those, 382 are E2-upregulated showing significantly higher *log*2 fold changes in expression than the rest (ERa-negative or compared to all; Wilcoxon-Mann-Whitney test p-value < 2.2e-16). In addition, ERa-positive long ncRNAs are significantly enriched amongst E2-upregulated cases (Fisher's exact test p-value = 3.237e-11, odds ratio = 1.63). For the 7,272 interacting genes (right): 1238 are ERa-positive and of those, 860 are E2-upregulated showing significantly higher *log*2 fold changes in expression (Wilcoxon-Mann-Whitney test p-value < 2.2e-16). In addition, ERa-positive genes are significantly enriched amongst E2-upregulated cases (Fisher's exact test p-value = 2.457e-11, odds ratio 1.39).

Supplementary Figure 4

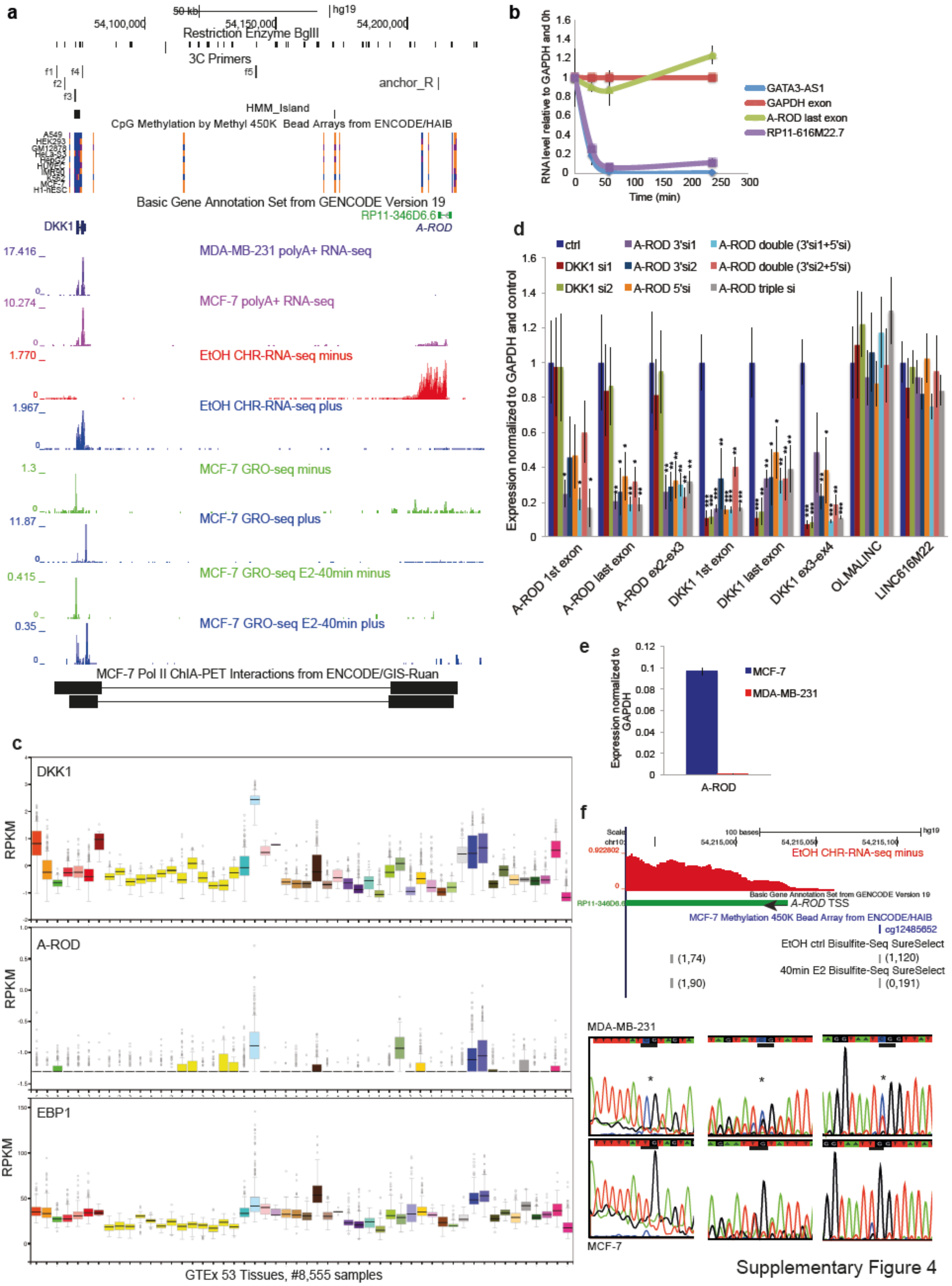

Supplementary Figure 4: A-ROD regulates DKK1 expression

(a) UCSC genome browser overview of the DKK1 and A-ROD (RP11-346D6.6) genomic loci. Location of the 3C primers, polyA+ RNA-seq data[5] from MDA-MB-231 and MCF-7 (GEO GSE37918), chromatin-associated RNA-seq in MCF-7 and GRO-seq[3] (GEO GSE43835) are shown (*y* axis reads per million). **(b)** RT-qPCR on whole-cell RNA from BrU pulse-chase triplicate experiments. MCF-7 cells where labeled with BrU for 30 minutes and then chased with excess of uridine for 30, 60 and 240 minutes. **(c)** Expression of DKK1, A-ROD and EBP1 (RPKM; plotted in the *log* scale for DKK1 and A-ROD) taken from the GTEx Project (version V6p). **(d)** Related to Fig. 3a. RT-qPCR levels of A-ROD, DKK1 and two unrelated long ncRNAs, in control and various siRNA treated cells, normalized to GAPDH and control condition. Whole-cell total RNA reverse-transcribed with random primers. Error bars represent normalized standard deviations from three independent experiments (n = 3 biological replicates, *P<0.05, **P<0.01, ***P<0.001, two-tailed Student's t-test). For each amplicon the statistical significance of the change in the siRNA treatment conditions is extracted by comparing to the respective control. **(e)** Whole-cell RNA RT-qPCR assaying expression of A-ROD in MCF-7 and MDA-MB-213 cells. A-ROD expression is not detected in MDA-MB-231 in agreement with polyA+ RNA-seq data from[5] (GSE37918) included as a track in a). **(f)** Upper panel: UCSC genome browser screenshot, zoom in the A-ROD promoter (±100 bp around TSS) showing ENCODE available DNA methylation data in MCF-7 (450K array assayed CpG, blue unmethylated; score = 97) and SureSelect bisulfite-seq (GEO submitted, Data Availability) in control (EtOH) and 40 min E2 treated MCF-7. There are two CpG's assayed, both unmethylated. In the parentheses, the number of methylated and unmethylated read counts per CpG is indicated (methylated, unmethylated). Lower panel: Sequencing electropherogram showing the methylation status of three CpG's from A-ROD promoter (PCR amplified fragment -300_+100 bp around TSS) in MCF-7 (lower track) and MDA-MB-231 (upper track). All three CpG's are unmethylated in MCF-7 (C is bisulfite-converted to T) but fully or partially methylated in MDA-MB-231 (protected from C>T conversion). The PCR of bisulfite-converted DNA was done as described in

Methods (Locus-specific DNA methylation analysis). The third CpG shown here was not assayed in the SureSelect system.

Supplementary Figure 5

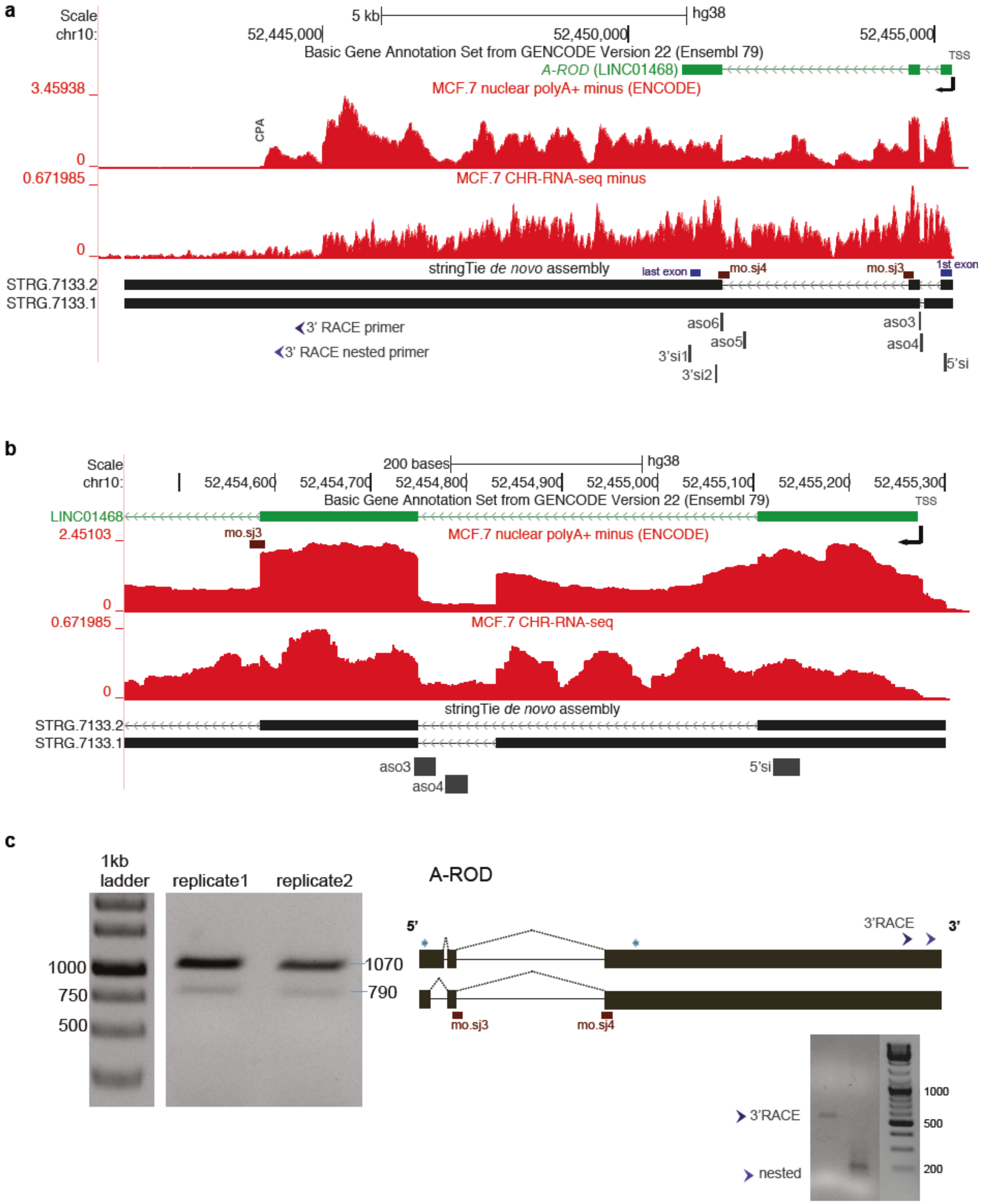

d

5′TTCTGTGCAAATTCTAATAAAAATGAAATGTAATTGATCCTCAAAAATATGATTCATCTATTTTAAGTCATCAGTC 3′

PAS blocker — CPA blocker

Supplementary Figure 5: Structure of the A-ROD transcript with siRNA and ASO positions.

(a) UCSC genome browser overview of the A-ROD transcription unit. Tracks of the ENCODE nuclear polyA+ RNA-seq and *de novo* transcript assembly from our total chromatin-asssociated RNA-seq (see Methods) are displayed. Blue bars depict first and last exon qPCR amplicons. **(b)** Zoom in the first exon-intron interval of A-ROD showing positions of the ASOs targeting the common first intronic sequence shared by the two isoforms. **(c)** Verification of the two predicted splicing isoforms by semi-quantitative PCR. The blue arrows above the scheme represent the left and right PCR primers (A-ROD_cDNA.F and A-ROD_cDNA.R, listed in Supplementary Table 1). The splicing-inhibiting morpholinos (Fig. 6) are represented by red bars: mo.sj3, targeting splice donor site of the 2nd intron; and mo.sj4, targeting the splice acceptor site. Position of the 3' RACE primers is also shown (Supplementary Table 1). Lower right panel: Analysis of the 3' RACE products on 1% agarose gel. The two bands were excised, DNA was gel-extracted and sequenced for 3' end verification (position of the cleavage and polyadenylation site, CPA). **(d)** Sequence around the CPA of A-ROD. Positions of the 2'OMePS oligos targeting that polyadenylation signal (PAS, the hexamer AATAAA) and the CPA and downstream T-rich stretch are underlined (related to Fig. 6).

Supplementary Figure 6

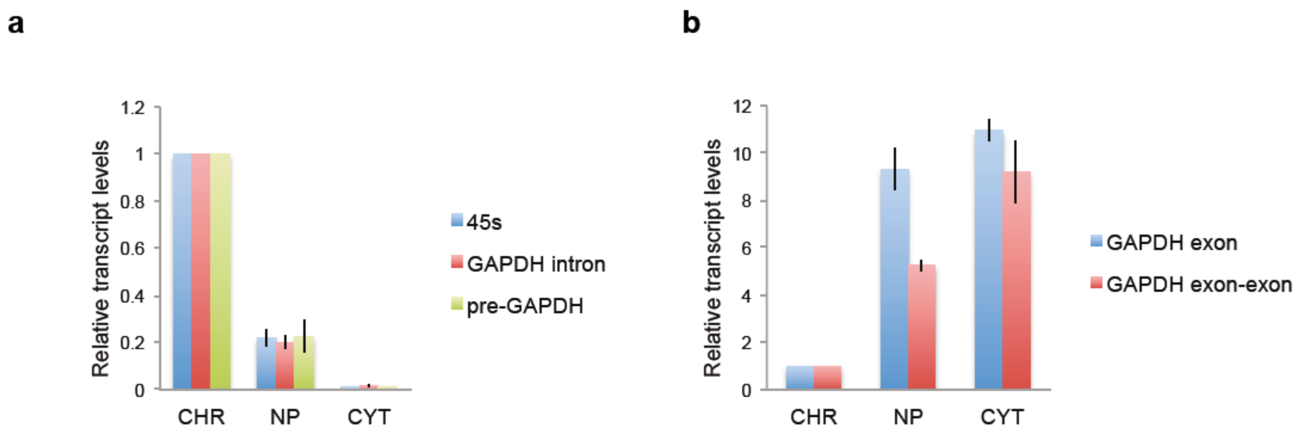

Supplementary Figure 6: Cell fractionation control

(a-b) RT-qPCR assaying the relative transcript levels of relevant markers in the three cellular fractions, normalized to the respective values in the chromatin-associated fraction. Error bars represent standard deviations from three independent fractionation experiments.

Supplementary Figure 7

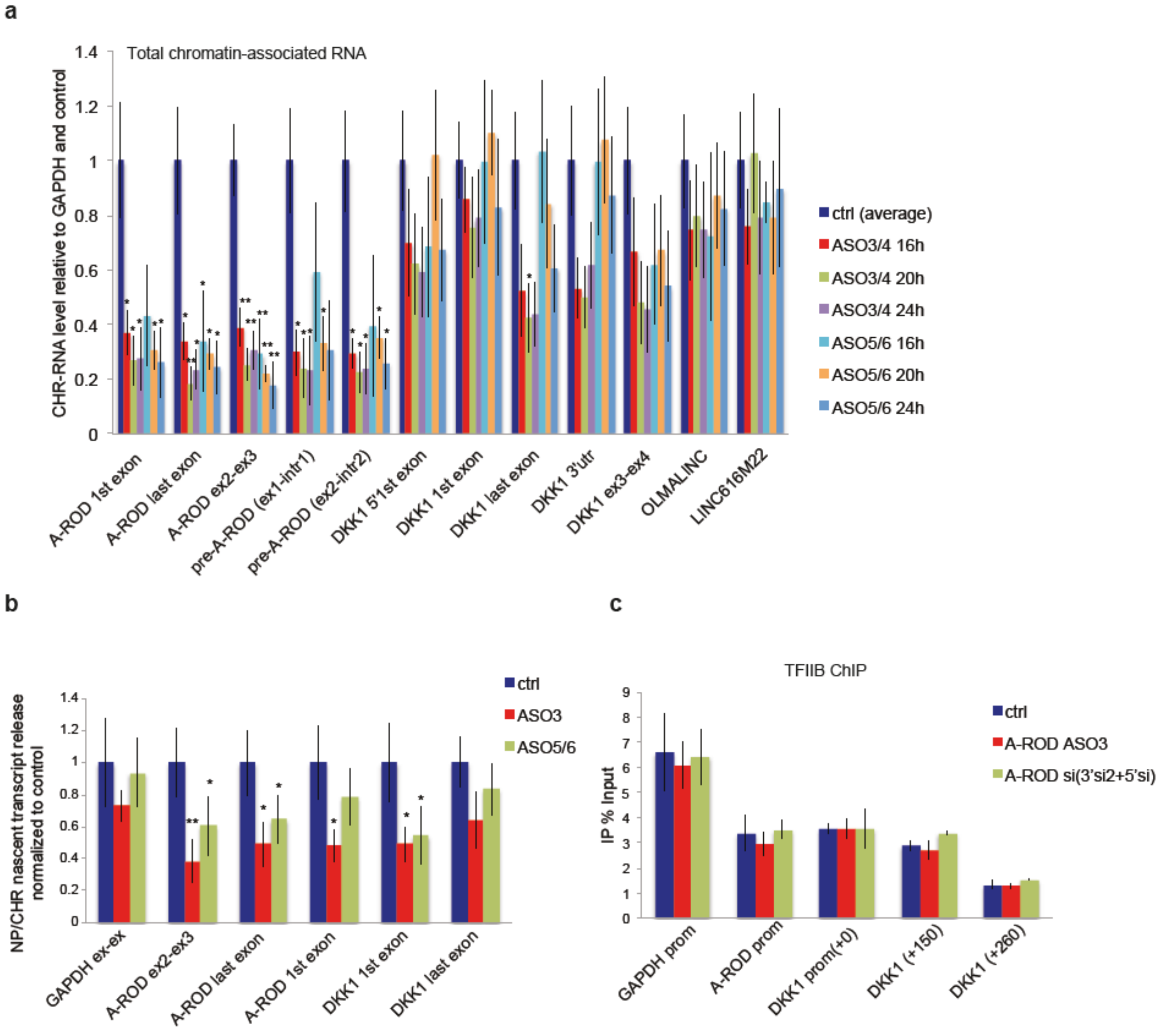

Supplementary Figure 7: A-ROD regulates DKK1 transcription at its release from chromatin

(a) RT-qPCR assaying the steady-state chromatin-associated RNA levels using several amplicons at three time-points post-transfection of pooled intronic ASO sequences at 100 nM final concentration; either ASO 3 and 4 targeting the first intron of A-ROD or ASO 5 and 6 targeting the second intron; or the standard negative control ASO. Error bars represent standard deviations from three independent experiments (n = 3 biological replicates, *P<0.05, **P<0.01, two-tailed Student's t-test). **(b)** Nascent transcript release assayed by extracting the ratio of BrU-labeled nucleoplasmic to

BrU-labeled chromatin-associated RNA. Error bars represent standard deviations from three independent experiments (n = 3 biological replicates, *$P<0.05$, **$P<0.01$, two-tailed Student's t-test). **(c)** TFIIB ChIP in control and A-ROD siRNA (here pooled A-ROD.5'si and A-ROD.3'si2) or A-ROD intronic-ASO3 treated cells. Error bars represent standard deviations from three independent experiments.

Supplementary Figure 8

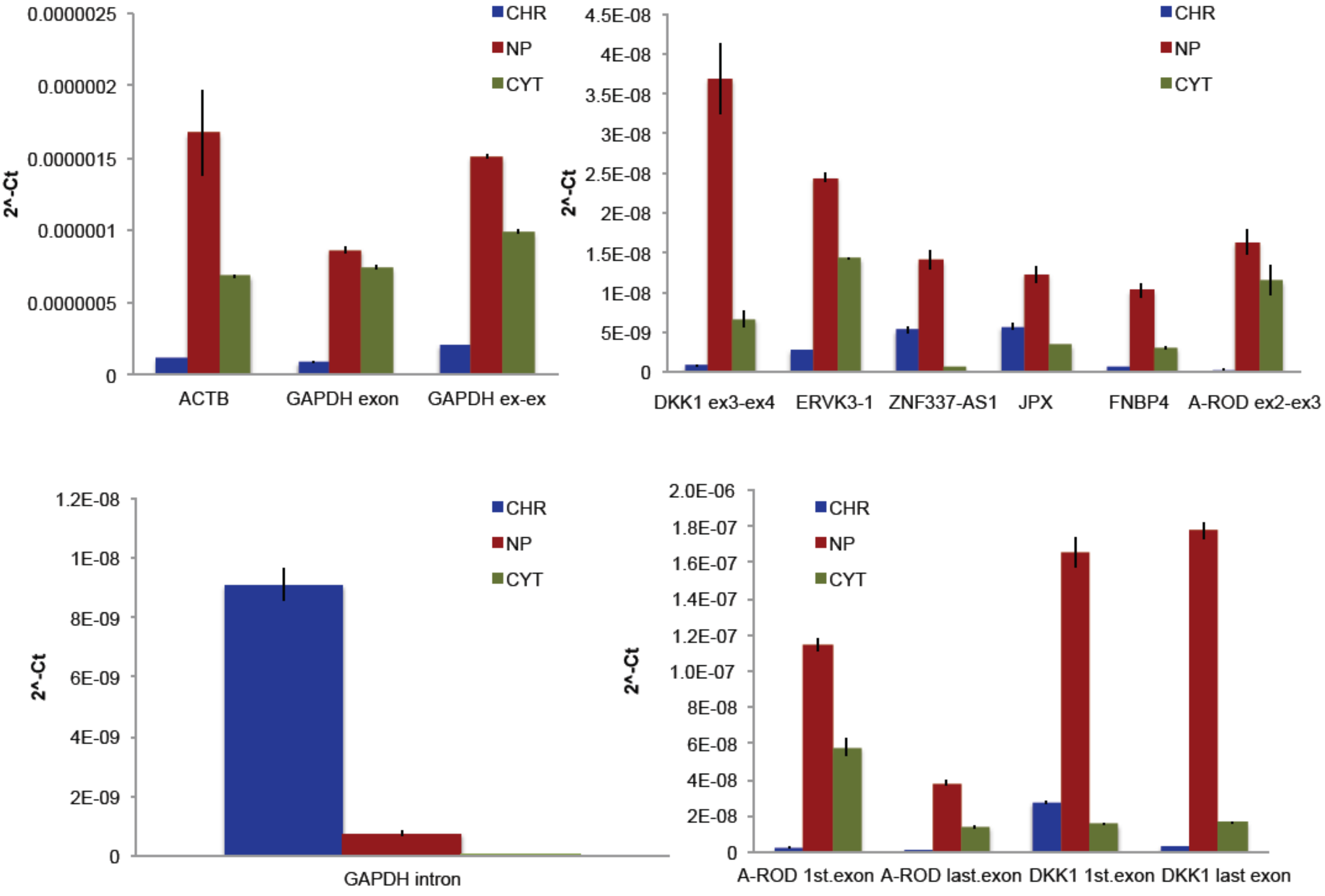

Supplementary Figure 8: Relative abundances of RNA transcripts in the three cellular fractions

RT-qPCR measured RNA levels upon cell fractionation. The absolute values (2^{-Ct}) are plotted. 300 ng of chromatin-associated (CHR), nucleoplasmic (NP) or cytoplasmic RNA (CYT) were reverse-transcribed with random primers. Error bars represent standard deviations from three independent fractionation assays.

Supplementary Figure 9

a

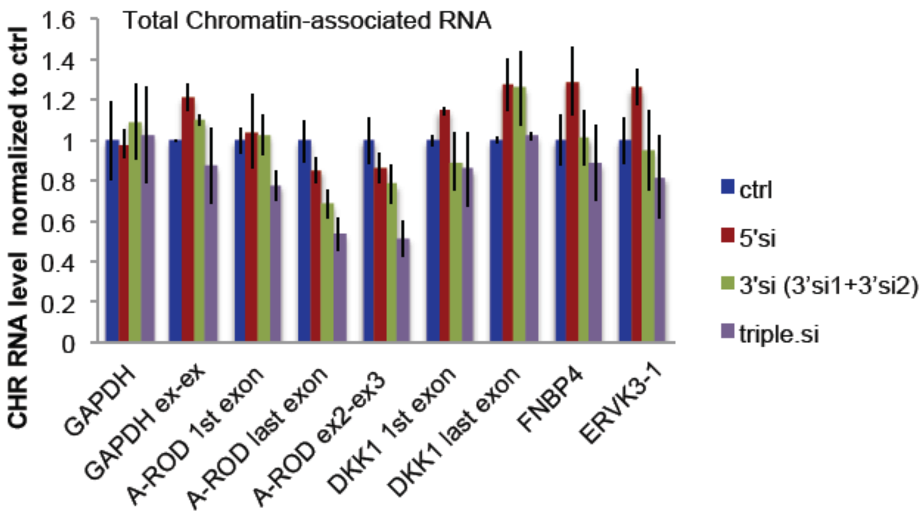

b

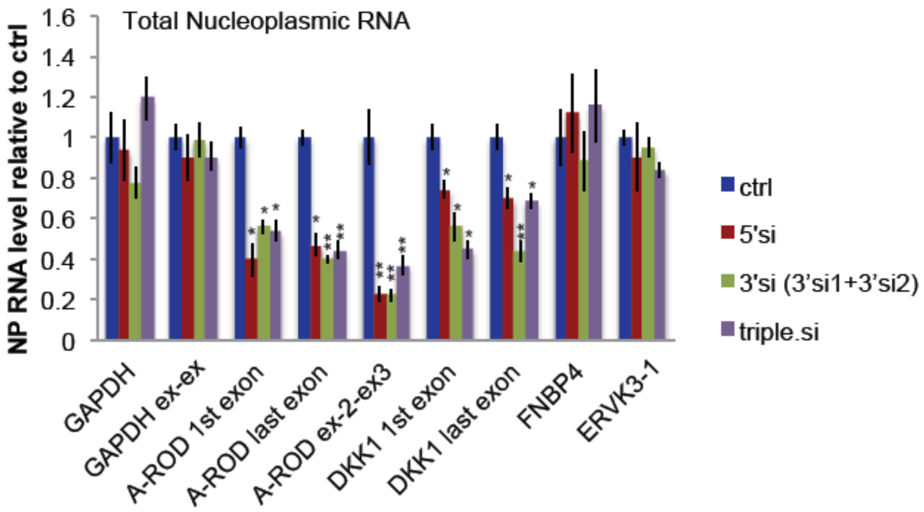

c

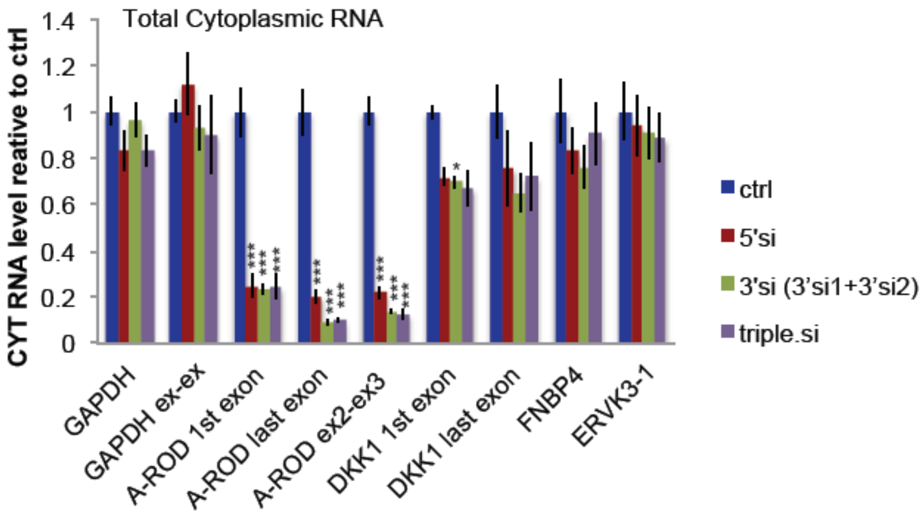

Supplementary Figure 9: RNA levels in the three cellular fractions upon siRNA transfection

(a-c) RT-qPCR measured RNA levels assessed in the three cellular fractions upon A-ROD siRNA transfection, normalized to the respective control. 300 ng of **(a)** chromatin-associated RNA, **(b)** nucleoplasmic RNA, and **(c)** cytoplasmic RNA were reversed-transcribed with random primers. The final siRNA concentration was kept constant at 45 nM (e.g. 15 nM of each siRNA in the triple transfection experiment). Error bars represent normalized standard deviations from three independent experiments (n = 3 biological replicates, *P<0.05, **P<0.01, ***P<0.001, two-tailed Student's t-test).

Supplementary Table 1. Primer sequences.

Primers used in RT-qPCR:	
GAPDH_prom.F	CGGTTTCTATAAATTGAGCCCGCAG
GAPDH_prom.R	CAAAAGAAGATGCGGCTGACTGTC
GAPDH_exon.F	GATTCCACCCATGGCAAAT
GAPDH_exon.R	GACAAGCTTCCCGTTCTCAG
GAPDH_mature.F	GCTCTCTGCTCCTCCTGTTC
GAPDH_mature.R	ACGACCAAATCCGTTGACTC
A-ROD_exon3.F	AAGGGGCAGCCAGTGAAGATG
A-ROD_exon2.R	TGGGGTTATCAGCAGTCTTCAAACT
A-ROD_prom.F	GTTCCCCTTCAGACAATCCTCATCG
A-ROD_prom.R	TTGTAGACTCTGAGTACGGTGCTGA
A-ROD_1st.exon.F	CTTGGCCTTTGTTCCCCTTC
A-ROD_1st.exon.R	TTGTAGACTCTGAGTACGGTGC
A-ROD_last.exon(body).F	CGTTCCACCTGCAAATACTATGCTC
A-ROD_last.exon(body).R	AGAGAAATGTCAACTTCTGGCCACA
DKK1_prom.F	GGCTTTGTTGTCTCCCTCCCAAG
DKK1_prom.R	TCACTTTGCAAGCCTGGGTCC
DKK1_5’1st.exon.F	CCTGACTCTGCAGCCGAACC
DKK1_5’1st.exon.R	AGCCATCATCTCAGAAGGACTCAAGA
DKK1_1st.exon.F	GGGTCTTTGTCGCGATGGTA
DKK1_1st.exon.R	GCGTTGGAATTGAGAACCGAG
DKK1_last.exon(body).F	CTTGCCGGATACAGAAAGATCACCA
DKK1_last.exon(body).R	AGTTCACTGCATTTGGATAGCTGGT
DKK1_3’utr.1F	AAGCATAACCCTTTACCCCATTT
DKK1_3’utr.1R	AGGTACCTATCATTTGTCATTCCAA
DKK1_3’utr.3F	TTTTCGGACAAGGAAGAAAATCATC
DKK1_3’utr.3R	TGTATTTATGGGCACACATCCTG
OLMALINC.F	TCCCCGATCTGCCCAACAATTTC
OLMALINC.R	GCCTGTCAGAACACCCACTCATT
LINC-616m22.F	CACCATCACTCGATTCCCAAAG
LINC-616m22.R	GTTTGCTCTAGGTCTGGGTCT
PA2G4F	AAAGAAGGCCTCCAAGACTGCAG
PA2G4R	CACCTCAGTCCCCAGCTTCATTT
A-ROD.readthrough1_F	AGTCTCCACATTGCTGACAGAACA
A-ROD.readthrough1_R	TGCTATGAATCAGGCACCACACT
A-ROD.readthrough2_F	TGACCTAACACTCCTCTGGTTCCT
A-ROD.readthrough2_R	CTGAAGTGGGGATGCAGGCA
A-ROD.readthrough3_F	AAACCCTAATCACAGCCAGTCCG
A-ROD.readthrough3_R	GCACAGTCTTCACAAAACAGCAACA
pre-A-ROD.F	TGATCTCACTCAGTCTCACGGCT
pre-A-ROD.R	TGAAAGGAGAGGTAGAGGCTGGAA
A-ROD.intronF	AGAAGCCAACATTACCCTAAGCCA
A-ROD.intronR	CCAGTTCCATTAGCCTGTGTCCA
ACTB_F	CCTGGCACCCAGCACAATGAA
ACTB_R	CGGACTCGTCATACTCCTGCTTGC
FNBP4_F	GAGATGCTGGCTGGCTGATA

FNBP4 _R	GCCTTGGAGGAAGGAGATGG
ZNF337-as1F	CCTTGCGTTCTCAGGTCTTTGGA
ZNF337-as1R	TCTGCCAGCACTAGGAGACAAGA
JPX_F	GGAAGACTTAAGATGGCGGCGTT
JPX_R	GGACTCATACTTCGGACGCCTTG
ERVK3_F	CGTGACAAGTGGTGCCCGAC
ERVK3_R	ATATTCTCCGCCGGCAGACTCTC
Primers used in semi-quantitative PCR (Supplementary Fig. 5C, left panel):	
A-ROD_cDNA.F	AATGACTTGTCTCGGTCAATTAGAGTA
A-ROD_cDNA.R	CCTGAGTCCTTGTAGACTCTGAGTAC
Primers used in 3' RACE (Supplementary Fig. 5C, right panel):	
A-ROD.last_exon.R 3'RACE	TCTCCTAGTAAGCCAGTAATGGAAAC
A-ROD.last_exon.pA_R nested	TGAACAAAGGGCAATGTAAGGCT
AUAP 3'RACE	GGCCACGCGTCGACTAGTAC
AP_RT 3'RACE	GGCCACGCGTCGACTAGTACTTTTTTTTTTTTTTTTTVN
Primers used in Fig. 6d:	
A-ROD_cDNA_RT	AGGAAGTTCCATAGCTAGTCTTCGTAG
A-ROD_cDNA_F2	GGGTTATCAGCAGTCTTCAAACTTG
A-ROD_cDNA_R1	AGATTGCACATCCAAGCCAAGATGCCA
Primers used in 3C:	
anchor_R	CCTAGCAGGGCTTGAGTCTCAACTCTGC
3c_f1	GAGCCACCATGCCTGGCCTCGATAGATC
3c_f2	CTAGCCTCTTAACTCCTTGGCACGATGC
3c_f4	GAAGGTTCTGTTTGTCTCCGGTCATCAG
3c_f3	GAGGAGGGCAACTGAAGGACCTCAAAGC
3c_f5	GAAAGTGGATATTTGCTACCATGCTTCA

Supplementary Table 1. Primer sequences.

Supplementary Table 2. Oligos for A-ROD purification; siRNA and ASO sequences.

A-ROD1	CTAACATCATCTTGAATTCTCC
A-ROD2	AGAGCAGTAGAGTTCTAAGTGG
A-ROD3	CATCCTTGCTCCCTCTGTGCCC
A-ROD4	AATCTCTTCTTCCCTTAGACTT
A-ROD5	TACTTGGCATCTTGGCTTGGAT
A-ROD6	CATCTGTAACAGAATGACTACC
A-ROD7	GGGCTCCGTCTTCAGACTTCTT
A-ROD8	TACCTCCATCTTCACTGGCTGC
A-ROD9	CAGAAGGGGCTCTTACTCTAGC
A-ROD10	ATAATTATCAGAGTTCCACTGG
A-ROD11	TCTCTGTTGAAAATTGTTCACA
A-ROD12	CTATGCTCCTTCATCTATATTA
A-ROD13	GTTCTATCAGCAGACAGATATA
A-ROD14	AGGCTTCTTTTGTAATTACTCA
A-ROD15	ACTATAATCTCTATTTCAGGGA
A-ROD16	GTCTGCACTGAGACATTCACTG
A-ROD17	TTGAGGCTGAAGATTAAGGGTC
A-ROD18	AGCACAGTAACTTATTATTAGC

A-ROD19	TATTATCACTGTACGTATTGTG
A-ROD20	CTGTGTTCAGGCGCTTGTGTAA
A-ROD21	AATCAACATGAATTTTGGTTGC
A-ROD22	TTTTGAAACTACATTGGGAGGA
LacZ_01	CCAGTGAATCCGTAATCATG
LacZ_02	GTAGCCAGCTTTCATCAACA
LacZ_03	ATCTTCCAGATAACTGCCGT
LacZ_04	ATAATTTCACCGCCGAAAGG
LacZ_05	TTCATCAGCAGGATATCCTG
LacZ_06	TGATCACACTCGGGTGATTA
LacZ_07	AAACGGGGATACTGACGAAA
LacZ_08	GTTATCGCTATGACGGAACA
LacZ_09	TGTGAAAGAAAGCCTGACTG
LacZ_10	GTAATCGCCATTTGACCACT
A-ROD.5'si_sense	rUrArCrUrGrCrGrArUrGrArGrGrArUrUrGrUrCrUrGrArAGG
A-ROD.5'si_antisense	rCrCrUrUrCrArGrArCrArArUrCrCrUrCrArUrCrGrCrArGrUrArGrC
A-ROD.3'si1_sense	rGrArCrCrGrArGrArCrArArGrUrCrArUrUrUrArArArCrUAA
A-ROD.3'si1_antisense	rUrUrArGrUrUrUrArArArUrGrArCrUrUrGrUrCrUrCrGrGrUrCrArA
A-ROD.3'si2_sense	rGrUrArGrGrArArGrUrUrCrCrArUrArGrCrUrArGrUrCrUTC
A-ROD.3'si2_antisense	rGrArArGrArCrUrArGrCrUrArUrGrGrArArCrUrUrCrCrUrArCrArC
DKK1.si1_sense	rArGrUrUrArArGrCrArUrUrCrCrArArUrArArCrArCrCrUTC
DKK1.si1_antisense	rGrArArGrGrUrGrUrUrArUrUrGrGrArArUrGrCrUrUrArArCrUrGrA
DKK1.si2_sense	rGrCrCrGrGrArUrArCrArGrArArArGrArUrCrArCrCrArUCA
DKK1.si2_antisense	rUrGrArUrGrGrUrGrArUrCrUrUrUrCrUrGrUrArUrCrCrGrGrCrArA
A-ROD ASO3	mC*mA*mU*CTG*TAA*CAGAA*TGACT*mA*mC*mC
A-ROD ASO4	mU*mU*mC*CAG*CCTCT*ACCTCT*CCT*mU*mU*mC
A-ROD ASO5	mA*mA*mC*ATT*ACCCT*AAGCCA*mU*mA*mA
A-ROD ASO6	mU*mG*mG*CTG*ATTCC*AAAGAT*ACA*mA*mG*mA
Standard Negative Control	mC*mG*A*CTA*TACG*CGCA*ATA*mU*mG*mG
* Phosphorothioate Bond, m = 2'-O-Methyl RNA base	
siRNA Negative Control Duplex: DS NC1 (IDT)	

Supplementary Table 2. Oligos for A-ROD purification; siRNA and ASO sequences.

References cited in the Supplementary Information

1. Corradin O*, et al.* Combinatorial effects of multiple enhancer variants in linkage disequilibrium dictate levels of gene expression to confer susceptibility to common traits. *Genome Res* **24**, 1-13 (2014).

2. Andersson R*, et al.* An atlas of active enhancers across human cell types and tissues. *Nature* **507**, 455-461 (2014).

3. Hah N, Murakami S, Nagari A, Danko CG, Kraus WL. Enhancer transcripts mark active estrogen receptor binding sites. *Genome Res* **23**, 1210-1223 (2013).

4. Welboren WJ*, et al.* ChIP-Seq of ERalpha and RNA polymerase II defines genes differentially responding to ligands. *EMBO J* **28**, 1418-1428 (2009).

5. Pellegrino L*, et al.* miR-23b regulates cytoskeletal remodeling, motility and metastasis by directly targeting multiple transcripts. *Nucleic Acids Res* **41**, 5400-5412 (2013).
