# Supplementary material for: Long ncRNA A-ROD activates its target gene DKK1 at its release from chromatin
Source: Nat Commun. 2018 Apr 24;9:1636. doi: 10.1038/s41467-018-04100-3 (PMC5915440; doi:10.1038/s41467-018-04100-3)
Supplement: Supplementary file 3 — Description of Additional Supplementary Files [file 41467_2018_4100_MOESM3_ESM.pdf]

Description of Additional Supplementary Files

File Name: Supplementary Data 1
Description: List of 4,467 long ncRNAs used for all bioinformatic analyses.

File Name: Supplementary Data 2
Description: Long ncRNAs significantly enriched at chromatin.

File Name: Supplementary Data 3
Description: Long ncRNAs significantly enriched in the nucleoplasm.
